# Book Review

**Published:** 1921-12

**Authors:** 


### Reviews of Books.

**The Psychology of the Special Senses and their Functional Disorders.** By ARTHUR F. HURST, M.A., M.D. Oxon., F.R.C.P. Pp. x., 123. London: Oxford Medical Publications. 1920. 12s. 6d. net.—The subject-matter of this book is taken from the Croonian Lectures for 1920, and is based on the text that hysteria is a condition in which symptoms are present which are the result of suggestion and are curable by psychotherapy. Dr. Hurst denies the presence of physical stigmata until they are looked for and produced by the physician, and regards suggestibility as the only mental factor necessary; and as this is a normal factor in everyone's mental make-up, anyone may develop an hysterical symptom provided the suggestion is strong enough. Interference with the functioning of the various senses may result from suggestions conveyed by the physician looking for stigmata, by perpetuation of an absence of perception during stupor or similar mental conditions, and perpetuation of absence sensation due to temporary organic lesions which have passed away. The immediate cause of the interference with function is the absence or overaction of attention in relation to the sensory area involved, and the scope of psychotherapy is to facilitate or inhibit the "attentive effort" towards these areas. This "attentive effort" is said to lower the resistance at synaptial junctions and its absence to increase this resistance, but the author does not venture to speculate as to what constitutes "attentive effort." The book is very readable, and the author's views on hysteria are clearly annunciated. All practitioners in psychotherapy will not find themselves in agreement, but the unification of all symptoms resulting from suggestion is not only convenient but pragmatically sound, since the early removal of hysterical symptoms is the ideal to aim at, however complicated a neurosis may be. The only risk of Dr. Hurst's method is that the inexperienced may fancy that, having removed the hysterical symptom, he has necessarily completed his duty to his patient.

**The Science of Ourselves.** By SIR BAMFYLDE FULLER, K.C.S.I., C.I.E. Pp. ix., 326. London: Oxford Medical Publications. 1921.—Sir Bamfylde Fuller covers a wide field and gives the outcome of long and varied experience. It is impossible here to summarise or to criticise the conclusions to which he has been led on an independent (sometimes rather too independent) line of thought. One must dig down to the fundamental issue. This is the old, yet ever new, problem of the relation of mind to body. It is commonly held that there are just two mutually incompatible solutions: (1) that mind is explicable in terms of certain very complex physiological events which occur in the brain or some part of it; (2) that mind belongs to an order of being disparate from that to which the body and the brain belong. When the author affirms that the development of the cerebral hemispheres "introduces a new and independent condition—that of consciousness," and when he says "that ideas, whether impression, record, or concept, are material things, and may be figured as clusters of brain-cells," he accepts the former solution, and he devotes the twenty-two chapters of this book to its elaboration. The alternative view—that which assumes the modern guise of either Vitalism or Animism—is scarcely so much as mentioned. But a third view is to-day striving to find expression in evolutionary terms. The author accepts three planes of evolution in the nervous system, and distinguishes them as the physical, the psychical, and the reflective, whereas we would extend this series. In the organism there are events on (say) five planes: (*a*) the physico-chemical; (*b*) the vital; (*c*) the instructive (in one sense of this word); (*d*) the unreflective; (*e*) the reflective. One never finds (*b*) without (*a*); (*c*) without (*b*) and (*a*); and so on. But why not frankly accept the events at any given level as something new which characterises that level?

**The Theory and Practice of Massage.** By BEATRICE M. GOODHALL-COPESTAKE. Third edition. Pp. xx., 270. London: H. K. Lewis and Co. 1920. Price 12s. 6d. net.—This is a very excellent and thoroughly practical treatise by an actual teacher who knows the difficulties which students find and the dangers which they have to avoid. It contrasts most favourably with certain former text-books in the omission of much unproved theory and of claims for wonderful results. The chief defect is that it is too brief and condensed, largely because the author has felt it necessary to include accounts of active and passive movements, general exercises, the re-education of muscles, and even of some exercising machines in her short volume. Recent experience has shown the absolute necessity of these additions to ordinary massage, but it is hardly possible to give useful teaching on them in fifty pages. There are excellent descriptions of the surgical and medical "conditions" which are treated by massage, with directions to the masseuse on her duty in every case. We should like to see in the section on rheumatoid arthritis, where, by the way, valvular disease of the heart is not common, emphasis laid on the great need of gentleness to avoid giving pain, and secondly the need of exercising patients on their feet when possible if the lower limbs are affected. When a patient takes to bed she is apt to become a prisoner for life.

**A Handbook of British Mosquitoes.** By WILLIAM DICKSON LANG, M.A., Sc.D. Pp. vii., 125. London: Printed by order of the Trustees of the British Museum. 1920. Price 20s. net.—The presence in England of large numbers of ex-soldiers infected with malaria parasites has of necessity focussed attention on the mosquito. Further, as *Anopheles Maculipennis* is widely distributed in these Isles, the importance of the association is self-evident. Another possible carrier, *A. Bifurcatus,* equally as common, together with *A. Plumbeus* complete the list of British Anopheles. Very clearly and simply the author points how to distinguish the sex, genus, and species of the gnat. To the majority, who may reasonably be contented to identify *A. Maculipennis,* the short account of the flies which may be mistaken for mosquitoes should be welcome. In Chapter iii. 21 species of mosquito are described in great detail. There is an interesting account of our present knowledge of the life-history of the mosquito. Even yet expert information is incomplete. We would have welcomed any observation from Dr. Lang on a related problem. Notwithstanding that we have in England the factors requisite for an outbreak of malaria, namely the presence of malaria-conveying Anopheles and individuals with parasites in the blood, yet the local infections in the British Isles have been few in number. The author throws cold water on the commonly-accepted belief that foreign mosquitoes can be introduced into this country by means of ships. The text is generously illustrated by 132 figures and 5 coloured plates. Many eminent living authorities have assisted Dr. Lang in several ways to produce a standard work of reference.

**Scopolamine-Morphine. Semi-Narcosis during Labour.** By WM. OSBORNE GREENWOOD, M.D. Pp. xi., 120. London: Oxford Medical Publications. 1918. Price 6s. net.—This little book gives an interesting history of the introduction of scopolamine-morphine semi-narcosis into obstetric practice, and an account of the results obtained by the earlier (mainly German) investigators. This part of the work, interesting though it is, is marred by the failure of the investigators to adopt any kind of standardisation for their observations. Collation of their results is therefore impossible. It should be stated that this failure is in no way due to the author, who has taken great pains to extract such information as could be got from the reports. The description of the chemistry of the alkaloids of hyoscyamus and belladonna is interesting, and gives useful information as to the isomeric forms in which they occur. The chapter on the objects of the treatment gives concisely the advantages of this method of treatment as opposed to the use of general anæsthetics, with the important warning that neither anæsthesia nor analgesia is the objective, but that the correct procedure aims at amnesia only. We are in entire agreement with the author on this point. The various methods advised by different investigators and clear directions as to the carrying out of the author's method are described under the head of "Technique." So far as the series of cases observed by the author is concerned, in which amnesia without analgesia was produced, such conditions as postpartum hemorrhage, uterine atony and delay in the second stage are shown to have been infrequent. We are not quite as confident that these conditions can be so generally excluded, but at the same time we consider that the frequency of their occurrence has been rather overstated. The effects on the child are more definite, but we doubt whether the author's contention that cases of still-birth could be prevented by this treatment is entirely justified. The author makes a good case for his method of administration and for the more general use of semi-narcosis. He emphasises the fact that haphazard administration and insufficient subsequent supervision of the patient are not without grave risks, and are likely to bring discredit on those who fail in these respects. The book is well arranged, clear and definite in detail, and gives at the end an interesting series of charts of cases treated by the author. We can recommend this book to those interested in the subject.

**Diathermy in Medical and Surgical Practice.** By Claude Saberton, M.D. Pp. xii., 138. Illustrated. London: Cassell & Co. Ltd. 1920.—The aim of this handbook is to serve as a guide to students and practitioners who wish to master the technique of diathermy. Short historical notes are followed by a chapter on the electrical principles of the apparatus' use, and then a short chapter follows on the construction of the principal forms of apparatus. Physical and physiological effects of diathermic currents, the methods of applying the current, lead up to the sections on the uses of diathermy in various pathological conditions. The information is concisely and clearly conveyed. The claims of diathermic methods are put forward, but are not overstated. The book seems to admirably fulfil its purpose, and affords a practical, short and reliable guide for beginners in the subject, and should prove of much value to general practitioners.

